# Individual and area-level factors associated with depression in Indonesia: a multilevel analysis using the 2018 national basic health research

**DOI:** 10.1186/s12889-025-23434-4

**Published:** 2025-10-02

**Authors:** Sri Idaiani, Muhammad Fiarry Fikaris, Gemala Chairunnisa, Mashita Fajri, Laura Anselmi, Jonathan Gibson, Irmansyah, Budi Anna Keliat, Ida Ayu Mas Amelia Kusumaningtyas, Asri Maharani, Aryana Satrya, Dwidjo Susilo, Matt Sutton, Hasbullah Thabrany, Herni Susanti, Helen Brooks, Penny Bee, Jack Wilkinson

**Affiliations:** 1 Research Centre for Preclinical and Clinical Medicine, National Research and Innovation Agency, Cibinong, Indonesia; 2 Graduate school of medical sciences, University of Groningen, Groningen, Netherlands; 3 Center for Health Economics and Policy Studies, Universitas Indonesia, Depok, Indonesia; 4 Faculty of Nursing, Universitas Indonesia, Depok, Indonesia; 5 Division of Population Health, Health Services Research & Primary Care, University of Manchester, Manchester, UK; 6 Research Centre for Public Health and Nutrition, National Research and Innovation Agency, Cibinong, Indonesia; 7 Division of Nursing, Midwifery & Social Care, University of Manchester, Manchester, UK; 8 Center for Social Security Studies, School of Strategic and Global Studies, Universitas Indonesia, Depok, Indonesia; 9 Department of Public Policy and Management, Faculty of Social and Political Sciences, Universitas Gadjah Mada, Yogjakarta, Indonesia; 10 National Commission for Tobacco Control, Jakarta, Indonesia

**Keywords:** Depression, Individual-level factors, District-level factors, National basic health research, Indonesia

## Abstract

**Background:**

Depression has become the leading cause of disease burden in low- and middle-income countries. However, evidence on the determinants of depression in those countries has been limited. This study aims to identify the factors in individual and area levels associated with depression using existing nationally representative data in Indonesia.

**Methods:**

Multilevel mixed-effects logistic regression models were performed on various national-scale Indonesian cross-sectional surveys and Indonesian Population Census to estimate those associations. We included adults aged 18 + who participated in the National Basic Health Research 2018 in this study. Depression was measured using the Mini International Neuropsychiatric Interview (MINI). Individual level variables include demographic characteristics, socioeconomic status, history of diseases, health behaviours, healthcare accessibility, and familial history of psychosis. District-level variables include the availability of health providers and professionals, regional gross domestic product, and the happiness index.

**Results:**

We found that individual-level factors, i.e., education, occupation, marital status, economic status, comorbidities, health behaviours, and difficulty with healthcare access were associated with the risk of depression. Happiness index as district-level factor, is related to the odds of depression. District-level factors, including the availability of general practitioners and mental health professionals and the density of healthcare providers, had no significant association with depression. The measured variables provided modest explanatory value overall.

**Conclusion:**

Individual-level factors are associated with depression among adults in Indonesia. Among the district level factors, only happiness index is related to depression. These results strengthen previous studies which stated determinants at the individual level are an important factor in depression. Therefore, effective prevention programs in mental health need to target both individuals and families.

**Supplementary Information:**

The online version contains supplementary material available at 10.1186/s12889-025-23434-4.

## Introduction

The growing evidence shows that mental disorders are becoming a significant burden in low- and middle-income countries (LMICs). Approximately 4.9% (95% Uncertainty Interval (UI) 3.9-6,1)) of Disability-Adjusted Life Years (DALYs) in 2019 were accounted for by various forms of mental and behavioural disorders [1]. Among mental disorders, depression stands out as the primary contributor to the burden of disease [1]. At the disorder level, depressive disorders were positioned as the 13th most prevalent out of the top 25 leading causes of DALYs globally in 2019 [1]. The majority of DALYs attributed to mental disorders (37·3% [95% UI 32·3–43·0]) were due to depression disorders, followed by anxiety disorders (22·9% [18·6–27·5]) and schizophrenia (12·2% [95% UI 6–15·2]). In 2019, the estimated prevalence of depression in Southeast Asia was 2610.6 cases per 100,000 population [1]. The prevalence of depression among individuals aged 15 + in Indonesia was 6.1% in 2018 [2]. These findings highlight the substantial contribution of depression to the overall burden of illness. Furthermore, severe depression often precipitates tragic events such as suicide attempts. Among individuals experiencing depression and suicidal ideation, approximately 10–15% ultimately take their lives [3].

In order to alleviate the burden of illnesses and suicidal ideation associated with depression, it is crucial to comprehend its underlying determinants and identify target groups for interventions. Depression stems from various physiological factors, including neurotransmitter imbalances in the central nervous system (CNS), disruptions in the hypothalamic-pituitary-adrenal (HPA) axis cycle, and genetic predispositions. These aspects require advanced diagnostic techniques such as polymorphism analysis or magnetic resonance imaging (MRI) to measure. However, diagnosing depression using these methods is difficult, costly, and time-consuming [3, 4]. Therefore, this research aims to identify the individual and area level factors associated with depression using existing nationally representative data in Indonesia.

Literature has reported factors associated with mental health disorders. Some of these studies examine the relationship between depression and socioeconomic factors, the GINI index, suicidal thoughts, demographics, access to health facilities, and other determinants [5–8]. The results of these studies generally state that individual factors play a greater role in depression than other contextual factors. However, unfortunately, there are some limitations in the studies related to depression, namely (1) most of the prior studies are in high-income countries [9]; (2) the ones in LMICs were not in nationally representative populations due to small sample size [9, 10]; (3) none to our knowledge looking at the nested structure of the area, a critical aspect when conducting analyses across different contextual settings in depression; and (4) limited studies look at how different dimensions of risk factors, such as demographics, socioeconomic, health behaviour, health status, and healthcare provision, are related to depression. As healthcare service provision has rested in the hands of local governments since decentralisation, the analysis of individuals’ health outcomes should not ignore the structure in which they are embedded within districts. In the case of hierarchically structured (nested) data, individuals belonging to the same districts are not truly independent; they are clustered as individuals sampled from the same district sharing common influences. Aggregation and disaggregation as used in the single-level model run the risk of ecological fallacy which may lead to bias in the analysis: what is true at one level need not be true at another [11, 12]. To address this bias, we used multilevel mixed-effects logistic regression models to recognise the nested nature of data by allowing for residual components at each level of the hierarchy [13].

Acknowledging the constraints of prior literature, we provide an analysis using existing data that examines the area-level factors linked to depression in Indonesia. Furthermore, considering the considerable inequality in healthcare including mental healthcare provision across the country [14, 15], the findings of this study could serve as evidence-based support for the development of future mental health programs.

## Data and methods

### Context

This study uses secondary data from several national-scale research studies conducted in Indonesia. Indonesia is an Upper Middle-Income country with a population of 270,625,567 people [16]. The government’s total expenditure on mental health was 2% of total government health expenditure. Total mental health professionals were 8135, consisting of 1120 psychiatrists with a ratio of 0.41 per 100,000 population, 6200 mental health nurses with a ratio of 2.29 per 100,000 population, 415 psychologists with a ratio of 0.15 per 100,000 population, 250 social workers with a ratio of 0.09 per 100,000 population and 150 other specialized mental health workers (e.g. Occupational Therapists) with ratio 0.06 per 100,000 population. Of the 34 provinces, 27 have mental health facilities, including 49 mental hospitals, 470 psychiatric units in general hospitals, and 150 community residential facilities. The remaining 7 provinces are without mental health hospitals [16]. Even though the majority of the population has National Health Insurance, mental disorder patients still have to cover 20% of their medical costs [16].

### Data sources

This study combines data from various sources linked by district identifier:

#### National basic health research 2018 (Riset Kesehatan Dasar or Riskesdas)

Riskesdas is a comprehensive health survey that has been conducted every five years since 2007 by the National Institute of Health Research and Development, Ministry of Health, Indonesia. This survey covers 34 provinces and 514 districts, aiming to provide nationally representative data on various health indicators. This study used Riskesdas 2018, which was conducted in 2018. 

#### National human resources for health survey 2017 (Riset Tenaga Kesehatan or Risnakes 2017)

Data from the 2018 Riskesdas was integrated with other data sources and statistics using district codes. The first linkage was with the 2017 Risnakes, which was conducted in 2017. Risnakes was the inaugural study to gather data on all health human resources in Primary Health Care Centres (PHCs or Puskesmas), public hospitals, and private hospitals across Indonesia. The Risnakes survey collected census data from 9,699 PHCs and 2,325 hospitals [17, 18]. The data are available for all districts and is representative at the district level.

#### Happiness level measurement survey (Survei Penilaian Tingkat Kebahagiaan or SPTK 2017)

Subsequently, the data was linked to the Happiness Level Measurement Survey (SPTK), which was carried out by the Central Bureau of Statistics (CBS) in 2017 across 34 provinces. This survey was representative at the provincial level [19]. The Happiness Index (HI) measurement tool was developed by BPS in 2012, and was first tested and implemented in a 2014 survey, using the NEF (New Economic Foundation) framework. The 2017 survey incorporated the OECD framework and used the Gallup World Poll method, allowing comparisons with international data [19]. Each indicator in the HI is assessed on a life satisfaction scale from 0 to 10, with 0 representing the lowest satisfaction and 10 the highest. A score of 5 is neutral, and the final value is multiplied by 10. An HI above 50 indicates better living conditions, while below 50 signifies increased unhappiness. The HI assesses three dimensions: life satisfaction, affect, and eudaimonia. Life satisfaction covers education, work, housing, income, health, family harmony, free time, social relations, environment, and security. The affect dimension includes happiness, lack of anxiety, and lack of depression. Eudaimonia involves independence, environmental control, self-development, positive relationships, life goals, and self-acceptance. Nineteen factors contribute to the HI.

#### Indonesian population census 2020

PHC density and Hospital density were calculated using the estimated population from the 2020 census.

Gross Domestic Regional Product (GDRP) data for 2018.

The GDRP per capita at the district level was obtained from Indonesia National Statistics.

### Data merging

Step 1: We used Riskesdas 2018 (National Basic Health Research) as our main source of individual-level data. Step 2: District and province codes in Riskesdas allowed us to link additional variables from four other data sources: Risnakes 2017, SPTK 2017, the 2020 Indonesian Population Census, and the 2018 GDRP estimates (Gross Domestic Regional Product). Where districts had been split or renamed, we used official references to unify them to current CBS codes (https://kodewilayah.id/). Step 3: We performed a series of many-to-one merges in Stata. First, we used the district code to merge district-level variables from Risnakes, SPTK, and the Census (for PHC and hospital density) to the individual-level Riskesdas data. Then, we merged the provincial-level GDRP data using the province code. Step 4: Records lacking a consistent code were flagged for review, and in instances of missing or unrecognisable codes, we excluded those observations.

### Measure of depression

Depression was evaluated using the Mini-International Neuropsychiatric Interview (MINI), version 6 of the International Classification of Diseases and Related Health Problems-10 (ICD-10) from Riskesdas 2018. This standardized interview is widely used to diagnose various mental health conditions and is available in multiple languages for use in clinical and research settings [20–22]. The depression section of the Indonesian version of the MINI has been validated in a previous study [23]. MINI, which is a structured diagnostic interview designed to assess mental disorders, including depression, using a decision-tree algorithm rather than a continuum scoring system. A respondent is classified as depressed if they answer “yes” to at least two core symptoms (items 1–3) and “yes” to at least two additional depressive symptoms (items 4–10) refer to Appendix 1). As one answer can have different symptom categorisation (core and additional), we could not use MINI as continuous variables.

#### Individual-level factors associated with depression

Depression-related individual-level factors were classified into demographic, socioeconomic, health behaviour, health status, and household characteristics. Demographic factors encompassed age (in years), but for this analysis we limited the sample to individuals over 18 years and sex (with males as the reference). Marital status was divided into single (reference), married, and divorced or widowed. Socioeconomic factors comprised education (primary school and lower, junior high school, and senior high school and higher as the reference), monthly per capita expenditure in quintiles, and occupation (employee and retirement as reference, students, unemployed, self-employed, informal workers, and others). Health behaviour determinants included smoking, tobacco use, alcohol use, and physical activities. Smoking behaviour was categorised into non-smoker (reference), ex-smoker, and smoker. Similarly, tobacco use behaviour was classified into non-user (reference), ex-user, and current user. The presence of chronic diseases was assessed using self-reports of diagnoses by health professionals, including tuberculosis, hypertension, stroke, diabetes, heart diseases, asthma, joint diseases, cancer, and renal failure. Other household characteristics included the presence of household members with psychosis and difficulty in accessing healthcare services. The difficulty in accessing healthcare services was calculated using principal component analysis (PCA) on three dimensions of information about accessing health facilities: (1) the modes of transportation used to access healthcare services; (2) the round-trip time from home to healthcare providers; and (3) the round-trip transportation cost to healthcare providers. The difficulty in accessing healthcare providers was categorised into easy (score ≥ mean/median) or difficult (score < mean/median) [24].

#### District-level factors associated with depression

To examine contextual effects, we utilized several factors that measure variations in local health provision across districts. These factors, pertaining to human resources for health, include the availability of (1) general practitioners in all PHCs; (2) mental health coordinators in all PHCs; (3) at least one clinical psychologist in any PHC; (4) psychiatrists in all hospitals; and (5) clinical psychologists in all hospitals. To gauge the availability of healthcare providers, we calculated the number of health facilities per 1,000 population, focusing particularly on PHCs and hospitals. As district-level determinants, we incorporated the Gross Domestic Regional Product (GDRP) per capita for 2018 and the happiness index surveyed in 2021. We merged the datasets using district identifiers published by CBS. We thus recoded the districts’ identifiers from the original data source to match the ones from the CBS.

### Statistical analysis

The analyses were designed to identify factors associated with depression. We fitted multilevel mixed-effects logistic regression models to estimate the association of individual and district-level factors with depression in Indonesia, treating the dependent variable as binary (having depression or not) while accounting for the nested structure of individuals within districts. The first level comprised individual characteristics, and district characteristics made up the second level.

For each predictor variable, we fitted a model including the variable with adjustment for age and sex [25, 26]. We adjusted the models by age and sex as they are plausibly prognostic of depression and are unlikely to lie on the causal pathway between any risk factor and outcome. We used a separate model for each variable to evaluate its association with depression while avoiding the Table 2 fallacy, which arises when we mistakenly try to interpret the mutually-adjusted coefficients from a multivariable regression model [27]. We considered not only the odds ratio (95% CI) for each predictor variable, but also the McKelvey and Zavoina (fixed and random effects) pseudo R-squared as a measure of explained variation [28]. We also conducted analyses to consider the predictive value of each of our groups of variables, as defined above. For this, we fitted six models including different sets of covariates: (1) null model; (2) a simple adjustment (age and sex); (3) adjustment as in Model 2 plus the adjustment for marital status and socioeconomic factors (education, per capita expenditure, and occupation); (4) adjustment as in Model 2 plus adjustment for health behaviours (smoking, tobacco use, alcohol use, and physical activities); (5) adjustment as in Model 2 plus adjustment for the presence of comorbidities, the presence of household members with psychosis and the difficulty in accessing healthcare services; (6) adjustment as in Model 2 plus adjustment for human resources for health, GDRP per capita, and happiness index; (7) adjustment as in Model 2 plus adjustment for health providers availability, GDRP per capita, and happiness index; (8) adjustment as in Model 2 plus socioeconomic factor, health behaviours, the presence of comorbidities, the presence of household members with psychosis, the difficulty in accessing healthcare services, human resources for health, GDRP per capita, and happiness index; and (9) adjustment as in Model 2 plus socioeconomic factor, health behaviours, the presence of comorbidities, the presence of household members with psychosis, the difficulty in accessing healthcare services, density of health providers, GRDP per capita, and happiness index. We considered the pseudo-R-squared, median odds ratio (MOR), and ICC (intra class correlation) as measures of explained variation.

We did not exclude variables based on multicollinearity concerns, as confounders are necessarily associated with exposures. Confidence intervals reflect a trade-off between information loss due to correlation and gain from adjusting for relevant variables. Omitting confounders would risk biased estimates, as discussed by Vanhove [29]. The statistical program used was Stata version 17.

## Results

There were 642,419 participants in the sample, of whom 38,615 (6%) were classified as depressed. Table 1 presents the characteristics of the sample in this study, which is divided into non-depressed (*n* = 603,804) and depressed (*n* = 38,615). On average, non-depressed and depressed participants were 42.34 (SD = 15.31) and 44.30 (SD = 16.32) years old, respectively. Most of the depressed individuals were females (63.68%) and graduated from primary school or had less educational attainment (55.93%). The highest proportion of those with depression were unemployed (38.24%), whereas among those without depression the highest proportion were informal workers (34.98%). The proportions of individuals with depression were higher among those in the lower per capita expenditure quintiles and those with comorbidities. 16.38% of respondents with depression had difficult access to healthcare services. This proportion is higher than those without depression (11.00%).

**Table 1:** Descriptive statistics on individual characteristics

No		No depression *n* = 603,804		Depression *n* = 38,615		*P* value
	Variables	*n* /mean	% /SD	*n*/mean	%/SD	
1a	Age (years), mean (SD)	42.33	15.31	44.29	16.32	< 0.001*
1b	Age Group, n (%)					
	18–24 years old	81,524	13.50	5,184	13.42	< 0.001
	25–34 years old	125,779	20.83	6,647	17.21	< 0.001
	35–44 years old	143,250	23.72	8,444	21.87	< 0.001
	45–54 years old	119,974	19.87	7,963	20.62	< 0.001
	55–64 years old	79,565	13.18	5,622	14.56	< 0.001
	65–74 years old	36,483	6.04	3,103	8.04	< 0.001
	75 years old and above	17,229	2.85	1,652	4.28	< 0.001
2	Sex					
	Male	290,676	48.14	14,025	36.32	< 0.001
	Female	313,128	51.86	24,590	63.68	< 0.001
3	Education					
	Senior high school and higher	229,460	38.00	10,515	27.23	< 0.001
	Junior high school	105,967	17.55	6,501	16.84	< 0.001
	Primary school and lower	268,377	44.45	21,599	55.93	< 0.001
4	Marital status					
	Single	93,084	15.42	5,760	14.92	< 0.001
	Married	453,463	75.10	26,606	68.90	< 0.001
	Divorced & Widowed	57,257	9.48	6,249	16.18	< 0.001
5	Occupation					
	Employee and retirement	75,720	12.54	2,604	6.74	< 0.001
	Student	17,585	2.91	1,191	3.08	< 0.001
	Jobless	171,728	28.44	14,765	38.24	< 0.001
	Self-employee	87,001	14.41	4,321	11.19	< 0.001
	Informal worker	211,224	34.98	13,202	34.19	< 0.001
	Others	40,546	6.72	2,532	6.56	< 0.001
6	Ever tuberculosis	2,803	0.46	479	1.24	< 0.001
7	Ever hypertension	51,025	8.45	6,004	15.55	< 0.001
8	Ever diabetes mellitus	12,750	2.11	1,734	4.49	< 0.001
9	Ever stroke	6,408	1.06	1,388	3.59	< 0.001
10	Ever asthma	14,988	2.48	2,256	5.84	< 0.001
11	Ever joint disease	48,477	8.03	6,261	16.21	< 0.001
12	Ever cancer	1,478	0.24	267	0.69	< 0.001
13	Ever heart disease	10,960	1.82	1,518	3.93	< 0.001
14	Ever renal failure	2,271	0.38	421	1.09	< 0.001
15	Smoking					
	Non-smoker	380,050	31.89	25,447	65.90	< 0.001
	Ex-smoker	31,186	5.16	2,551	6.61	< 0.001
	Smoker	192,568	31.89	10,617	27.49	< 0.001
16	Tobacco use					
	Non-user	570,017	94.40	35,111	90.93	< 0.001
	Ex-user	6,089	1.01	708	1.83	< 0.001
	User	27,698	4.59	2,796	7.24	< 0.001
17	Alcohol					
	Not at all	575,783	95.36	36,125	93.55	< 0.001
	Under standard	16,384	2.71	1,446	3.74	< 0.001
	More than standard	11,637	1.93	1,044	2.70	< 0.001
18	Physical activity					
	Active	544,302	90.15	34,502	89.35	< 0.001
	Less active	59,502	9.85	4,113	10.65	< 0.001
19	Economic status					
	Quintile 5 (the wealthiest)	137,905	22.84	6,772	17.54	< 0.001
	Quintile 4	127,100	21.05	7,675	19.88	< 0.001
	Quintile 3	119,300	19.76	8,051	20.85	< 0.001
	Quintile 2	113,177	18.74	8,044	20.83	< 0.001
	Quintile 1 (the poorest)	106,322	17.61	8,073	20.91	< 0.001
20	Have psychosis in HH					
	No	599,061	99.21	37,822	97.95	< 0.001
	Yes	4,743	0.79	793	2.05	< 0.001
21	Access to Healthcare					
	Not difficult	484,453	89.00	27,957	83.62	< 0.001
	Difficult	59,884	11.00	5,476	16.38	< 0.001

* t test, the rest used chi square

This study covered all 514 districts in Indonesia. The numbers of districts with general practitioners and mental health coordinators in all PHCs are 326 (63.42%) and 294 (57.19%), respectively (Table 2). Only one district had a clinical psychologist in at least one PHC. The proportion of districts with psychiatrists and clinical psychologists in all hospitals was 6.03% and 5.83%, respectively. Psychiatric hospitals were available in 414 (80.54%) districts. On average, a district had 6.39 (SD = 5.00) PHCs and 1.07 (SD = 0.92) hospitals. The mean GDRP per capita per district is $1.25 (SD = 2.72), while on average, the happiness index in each province was 71.01 (SD = 1.53).

**Table 2:** Descriptive statistics for district characteristics (*n* = 514)

	Variables	*N* or mean (SD)	District			City				*p*	
			n/mean	%/SD	n/mean			%/SD			
1	All PHCs in the district have GP	326	233	71.47	93		28.53		< 0.001		
2	All PHCs in the district have a mental health coordinator	294	226	76.87	68		23.13		0.007		
3	At least one PHC in the district has a clinical psychologist	1	0	0	1		100		0.039		
4	All hospitals in the district have a psychiatrist	31	30	96.77	1		3.23		0.021		
5	All hospitals in the district have a clinical psychologist	20	20	100	0		0		0.027		
6	Psychiatric hospital available	414	325	78.50	89		21.50		0.004		
7	Density of PHC, mean (SD)	6.39 (5.00)	6.93	5.28	4.09		2.49		< 0.001*		
8	Density of hospital, mean (SD)	1.07(0.92)	0.78	0.56	2.17		1.19		< 0.001*		
9	GRDP at current market prices 2018, mean (SD)	$1.25(2.72)	$0.89	$1.41	$2.73		$5.28		< 0.001*		
10	Happiness Index 2017, mean (SD)	71.01 (1.53)	70.95	1.74	71.22		1.64		0.166*		

Table 3 shows the association between the odds of depression and each of the individual and district-level factors adjusted for age and sex. Participants who were married had 24% lower odds of being depressed than those who were single (OR = 0.76, 95% CI: 0.74–0.79). The odds of being depressed were higher among participants who were divorced and widowed than those who were single (OR = 1.16; 95% CIs = 1.10 to 1.22). Having higher education attainment and being wealthier were associated with lower odds of depression. Occupation status had a significant relationship with depression, with students and unemployed participants having higher odds of depression than those other types of occupation compared to employed and retired participants. Having higher per capita expenditure was associated with a lower risk of depression. The presence of comorbidities was linked to higher odds of depression. Past smokers and active smokers had 1.81 (95% CIs = 1.72 to 1.91) and 1.52 (1.47 to 1.58) higher odds of depression. Not consuming alcohol and having active physical activity were associated with lower odds of depression. The presence of household members with psychosis had a significant and positive association with depression (OR = 2.72; 95% CIs = 2.52 to 2.95%). Those who reported difficult access to healthcare had 1.55 (95% CIs 1.50–1.60) higher odds of depression.

Turning to the district-level variables, the availability of general practitioners and mental healthcare professionals and the density of healthcare providers were not associated with depression, with reasonably precise estimates containing the null value. The median odds ratios of the models varied slightly between 2.084 and 2.151, suggesting that no one factor, in combination with age and sex, was sufficient to explain variation between areas. Neither the ICC nor pseudo-R2 values were substantially different from those obtained from a model that included age and sex only (Table 3), suggesting a limited explanatory value for each predictor variable.

**Table 3:** Multilevel mixed effects ordered logistic regression results on the associations between each of the risk factors and depression among Indonesian adult

	Variables	OR (95%CI)	*P* value	ICC	Pseudo R2	MOR
1	Education					
	Senior high school and higher	reference		0.164	0.173	2.151
	Junior high school	1.40 (1.35–1.44)	< 0.001			
	Primary school and lower	1.75 (1.71–1.80)	< 0.001			
2	Occupation					
	Employee and retirement	reference	< 0.001	0.160	0.171	2.128
	Student	2.07 (1.93–2.23)	< 0.001			
	Jobless	1.97 (1.88–2.06)	< 0.001			
	Self-employee	1.45 (1.38–1.52)	< 0.001			
	Informal worker	1.78 (1.71–1.87)	< 0.001			
	Others	1.52 (1.44–1.61)	< 0.001			
3	Marital status					
	Single	reference		0.160	0.164	2.128
	Married	0.76 (0.74–0.79)	< 0.001			
	Divorced & Widowed	1.16 (1.10–1.22)	< 0.001			
4	Economic Status					
	Quintile 5 (the wealthiest)	reference		0.161	0.167	2.136
	Quintile 4	1.27 (1.23–1.32)	< 0.001			
	Quintile 3	1.42 (1.36–1.47)	< 0.001			
	Quintile 2	1.53 (1.48–1.58)	< 0.001			
	Quintile 1(the poorest)	1.64 (1.58–1.70)	< 0.001			
5	Ever tuberculosis	2.71 (2.45-3.00)	< 0.001	0.160	0.162	2.127
6	Ever hypertension	1.73 (1.67–1.78)	< 0.001	0.159	0.165	2.123
7	Ever diabetes mellitus	1.93 (1.83–2.04)	< 0.001	0.159	0.163	2.125
8	Ever stroke	3.24 (3.04–3.44)	< 0.001	0.160	0.165	2.128
9	Ever asthma	2.24 (2.14–2.35)	< 0.001	0.158	0.165	2.120
10	Ever heart disease	1.97 (1.87–2.09)	< 0.001	0.159	0.163	2.125
11	Ever joint disease	2.09 (2.02–2.15)	< 0.001	0.160	0.169	2.133
12	Ever cancer	2.36 (2.06–2.71)	< 0.001	0.159	0.162	2,127
13	Ever renal failure	2.68 (2.40–2.98)	< 0.001	0.159	0.162	2.126
14	Smoking					
	No smoker	reference		0.156	0.167	2.107
	Ex-Smoker	1.81(1.72–1.91)	< 0.001			
	Smoker	1.52(1.47–1.58)	< 0.001			
15	Tobacco use					
	Not at all	reference		0.159	0.162	2.123
	Ex tobacco user	1.57(1.45–1.70)	< 0.001			
	Tobacco user	1.37(1.30–1.43)	< 0.001			
16	Alcohol					
	No alcohol	reference		0.157	0.164	2.112
	Below threshold	1.63 (1.54–1.73)	< 0.001			
	More than threshold	1.55 (1.45–1.67)	< 0.001			
17	Physically active	0.93 (0.89–0.96)	0.003	0.159	0.161	2.127
18	The presence of household members with psychosis	2.72 (2.52–2.95)	< 0.001	0.160	0.163	2.128
19	Have difficult access to healthcare	1.55(1.50–1.60)	< 0.001	0.156	0.159	2.104
20	Puskesmas density	1.00 (0.98–1.01)	0.820	0.159	0.161	2.127
21	Hospital density	1.02 (0.94–1.10)	0.563	0.153	0.159	2.087
22	All PHC in the district have GP	0.97(0.84–1.13)	0.772	0.159	0.163	2.126
23	All PHC in the district have mental health coordinator	0.94(0.82–1.08)	0.430	0.160	0.163	2.125
24	At least one PHC in the district has a clinical psychologist	1.13 (0.23–5.44)	0.878	0.159	0.163	2.127
25	All hospital in the district have psychiatrist	1.23 (0.92–1.65)	0.158	0.159	0.161	2.124
26	All hospital in the district has clinical psychologist	1.05 (0.74–1.49)	0.779	0.153	0.160	2.084
27	Availability Mental Hospital in province	0.85 (0.71–1.02)	0.088	0.158	0.161	2.121
28	GDRP 2018	1.00 (0.99-1.00)	0.314	0.159	0.161	2.126
29	Happiness Index 2017	1.05 (1.01–1.09)	0.009	0.158	0.161	2.117

Note: * Adjusted with age group and sex.

Results of analyses examining the explanatory value of each group of variables are shown in Table 4. The MOR was 2.125 in the empty model. The value increased slightly to 2.127 when age and sex were included in the analysis. The highest MOR (2.149) was in Model 3 when we added socioeconomic factors. It shows that in the median case, the residual heterogeneity between districts increased by 2.149 times the individual odds of having depression. The MORs decreased in the next models. Similar patterns were seen in the ICC, which showed models decreases as groups of variables were added. Model 3 had the highest ICC (0.164). It showed that the proportion of total variance in the odds of depression attributable to the district level was 16.4%. The pseudo-r2 suggested that the measured variables provided relatively limited explanatory value, which increased slightly as we added more variables in the analysis, from 0.143 in the null model to around 0.207 and 0.203 in Models 8 and 9 adjusted with all individual-level and district-level factors.

**Table 4:** Measures of variation and model fit for each multilevel mixed-effects ordered logistic regression model predicting depression among Indonesian adults

Variable group	Intra-class correlation	Pseudo-r2	Median odds ratio	Missing data
*Model 1* (Empty model)	0.160	0.143	2.125	33 (0.005%)
*Model 2* (Age and sex)	0.160	0.161	2.127	33 (0.005%)
*Model 3* (Model 2 + socioeconomic factors)	0.164	0.184	2.149	33 (0.005%)
*Model 4* (Model 2 + health behaviours)	0.153	0.170	2.092	33 (0.005%)
*Model 5* (Model 2 + comorbidities + the presence of household members with psychosis + difficult healthcare access)	0.154	0.174	2.093	64.682 (10.02%)
*Model 6* (Model 2 + human resources for health + GRDP per capita + happiness index)	0.156	0.161	2.109	33 (0.005%)
*Model 7* (Model 2 + health providers availability + GRDP per capita + happiness index)	0.150	0.159	2.070	29.190 (4,54%)
*Model 8* (Model 2 + socioeconomic factors + health behaviours + comorbidities + the presence of household members with psychosis + difficult healthcare access + human resources for health + GRDP per capita + happiness index)	0.149	0.207	2.066	64.682 (10.02%)
*Model 9* (Model 2 + socioeconomic factors + health behaviours + comorbidities + the presence of household members with psychosis + difficult healthcare access + health providers availability + GRDP per capita + happiness index)	0.142	0.203	2.022	85.885 (13.36%)

(Note: socioeconomic factors = marital status, education, occupation and wealth (in quintiles); health behaviour = smoking, alcohol and physical activity; comorbidities = the presence of diseases, including tuberculosis, hypertension, stroke, diabetes, heart disease, cancer, renal failure, asthma, joint disease; human resources of health = availability of general practitioners (GP) in primary healthcare (PHC), mental health project manager in PHC, clinical psychologists in PHC, clinical psychologists in hospital, and psychiatrists in hospital; health providers availability = density of PHC, density of hospital, mental hospital availability; GRDP = Gross Regional Domestic Product)

## Discussion

This study aims to address research gaps by examining how different groups of variables measured at the individual or district level are associated with depression using nationally representative datasets from Indonesia. Referring to that aim, this analysis suggested that depression was associated with individual-level factors, i.e., education, occupation, marital status, economic status, comorbidities, health behaviours, and difficulty with healthcare access.

District-level factors were not clearly associated with depression. A cross-national study comparison found that while individual socioeconomic status played a stronger role in determining depression risk, country-level economic disparities had a limited effect, particularly in more developed nations [30]. Similarly, a study by Lund et al. demonstrated that poverty is a significant risk factor for common mental disorders, but macroeconomic indicators such as GDP were not consistent predictors of individual mental health outcomes. These findings align with our results, suggesting that while healthcare accessibility and economic development at the district level are relevant, individual socioeconomic conditions remain the strongest predictors of depression risk in Indonesia [31]. Although factors associated with depression were identified, the explanatory value of the studied factors, either alone or in combination, was reasonably modest. Consequently, measures of explained variation at both the individual and district levels suggested that national variation in depression remains largely unexplained. While individual and area-level factors are associated with depression, they do not provide a fully predictive model for diagnosing individuals. The MINI, as a structured diagnostic interview, directly identifies depression cases, emphasizing the need for systematic screening rather than relying solely on risk factor-based inference.

We found slight differences in pseudo-R2 when we added district-level factors in the analysis compared to models with only individual-level factors (Table 4), suggesting that those factors provided limited explanatory value. These results are in line with previous research on depression with multilevel analysis, which states that depression is influenced by the individual level, not other contextual factors [5, 6, 30, 32, 33]. Among these individual factors, socioeconomic factors are most frequently identified as contributing factors, in line with our finding that the median odds ratio in Tables 3 and 4 is highest when socioeconomic status is included. The implication is that people will be vulnerable to depression if they have poor socio-economic conditions.

Socioeconomic factors, specifically economic status and GDRP, serve as proxies, but GDRP does not have a significant role. Both GINI and GDRP are reflections of macroeconomic status so they are not always relevant in describing individual economic status. However, a study of birth mortality using multilevel analysis shows that the GINI ratio is related to mortality output [34]. Some use the term income deprivation, namely as the percentage of households in each enumeration district with gross household income of less than £ 10,000 per annum [35]. That study also stated that income deprivation was related to depression in addition to social cohesion. Residing in a prosperous country or region does not necessarily safeguard an individual from depression. The individual’s income level, or the amount of money they earn, plays a more crucial role. Income inequality can pose a risk to an individual’s mental health. The greater the income disparity in a country or region, the higher the risk of depression among the population [30, 36]. Conversely, individuals from low socioeconomic groups residing in areas or countries with low economic levels may experience a deterioration in their mental health. Even though individual factors are predominant, residing in impoverished surroundings can have a detrimental effect [36]. In fact, several studies on depression and individual socio-economics are more specific in classifying assets and expenditures in socioeconomic terms [31]. This aligns with the findings of this research, which suggest a relatively higher (compared to other factors considered) increase in pseudo R2 when economic factors are incorporated as in Model 3.

We found that living in a province with higher happiness index is related to risk of having depression (OR = 1.05; 95% CI = 1.01; 1.09). This finding appears to be counter-intuitive. However, we note that the results do not imply any causal association between happiness and depression. For example, the happiness index is a group-level predictor and does not indicate the state of particular individuals in the survey. Moreover, we present minimally-adjusted estimates, such that an omitted variable may explain the association. Finally, we note that this study uses cross-sectional data, which precludes evaluation of causal effects.

Several countries have implemented innovative approaches to tackling depression among populations with low socioeconomic conditions. The UK’s Improving Access to Psychological Therapies (IAPT) program provides free, evidence-based psychological treatments [37, 38]. While the program has successfully expanded access to therapy, long waiting times remain a challenge. In Indonesia, BPJS Kesehatan, the national health insurance scheme, covers mental health services, but availability remains limited, especially in rural areas. Expanding community-based mental health services through Puskesmas could improve accessibility and feasibility. Another approach is integrating mental health support into financial assistance programs. Brazil’s Bolsa Família, a conditional cash transfer (CCT) program, has indirectly reduced stress and improved mental well-being among low-income families by providing financial relief [39]. Similarly, Indonesia’s Program Keluarga Harapan (PKH) supports low-income households, but it does not explicitly address mental health [40]. A feasible step forward would be incorporating mental health education and psychological support into PKH to address the psychosocial stressors faced by beneficiaries. While international experiences provide valuable insights into policies that address depression among low-SES populations, Indonesia must adapt these models to fit its unique social, economic, and cultural landscape.

The findings of this study align with the classical theory, which posits that women are more susceptible to depression than men, individuals with lower education are more vulnerable than those with higher education, and a separated marital status poses a risk for depression [3, 4, 41]. Women are often associated with reproductive cycle while other conditions are conditions where a person’s ability to deal with stress is poor [42]. Specifically, marital status is largely influenced by personal circumstances [42].Individuals who are divorced or have lost their partner are likely to live alone, which can increase their vulnerability to depression due to having a partner as a form of social support [43].

Chronic illnesses have consistently been included in previous studies as a determinant of depression [44]. The underlying mechanism of the relationship between depression and chronic illness is that people with chronic diseases tend to have negative interpretations of themselves [45]. Another mechanism suggests that restrictions on physical activity in people with chronic diseases will lead to disappointment [46]. In this study, these diseases were identified through self-reported having diagnosed by health professionals in interviews rather than physical and laboratory examinations, yet they demonstrated an association with depression. Other diseases and risk factors, such as multiple sclerosis, epilepsy, and obesity, also have association with depression [44]. Chronic diseases increasingly influence the older people [47]. This study highlights the role of other individual factors such as tobacco use and alcohol consumption. These variables are seldom found in other studies due to the issue of sample adequacy, as it is challenging to find a population with high alcohol consumption [44]. In Indonesia, alcohol consumption is predominantly found in North Sumatra and Eastern Indonesia [2]. Addictive substances like tobacco, alcohol, and illegal drugs are almost certainly negative determinants of mental health hence they are often included in depression studies.

In this study, access to health facilities also plays a significant role and acts as a negative determinant of disease. One of the plausible explanation is that long distances to health facilities pose challenges in accessing diagnosis and treatment [5]. Furthermore, Indonesia still has a low ratio of health workers and disparities in health facilities. The health facilities inquired about by survey respondents in Indonesia were PHCs and hospitals. The density of PHCs and hospitals in this study did not significantly contribute to depression, suggesting that the issue is not solely the quantity but also that the health programs provided have not yet reached the community. This issue is exacerbated by the inequality of health facilities, particularly in Eastern Indonesia [2].

Literature suggests that physical activity has a more significant impact if the degree of depression is severe, while its relationship with mild and moderate depression is not as pronounced [48]. Other literature states that in adults, physical activity reduces depression if they do brisk walking for at least 2.5 h a week [49]. In this analysis, it appears that physical activity does not have an association with depression. Furthermore, in model 4, health behaviours appear to explain relatively little of depression risk. It is important to note that, according to the 2018 Riskesdas, a very small proportion of the Indonesian population engages in sufficient physical activity. This factor should be considered when interpreting the results of this study, as the Riskesdas data only measures the presence or absence of depression in individuals without assessing the degree of depression [2].

Other determinants that could have been analysed in this study include population density, but this was not possible due to the lack of information on area per region at the year of the study. Literature suggests that population density impacts depression [50]. However, due to the vast area of Indonesia and the disparities in population density, this factor was not evaluated in this study. Consequently, environmental factors such as security, crime, and violence are represented solely by cohabitation with an individual suffering from psychosis. It is known that many individuals with psychosis do not receive regular treatment, either due to lack of knowledge, attitudes and family behaviour, or access to healthcare. The condition of untreated individuals can affect the wellbeing of the family living in the same household.

One of the limitations of this study is that the analysis used cross-sectional datasets. Therefore, this study could not provide an estimate of the causal determinants of depression. Instead, these analyses highlight how individual and district-level characteristics are associated with current depression status. Another limitation is that the analysis was limited to variables available within the 2018 National Basic Health Survey and related national datasets. As such, we were unable to account for several potentially important determinants of depression, including biological and genetic factors, central nervous system functioning, comorbid medical conditions, quality of life, social support, social isolation, stressful life events, and health behaviours such as gambling [3, 4, 41, 51, 52]. These unmeasured variables may confound or mediate the observed associations and should be considered in future research using more comprehensive datasets. Third, the analysis did not employ spatial-temporal modelling, which may have further enriched the understanding of geographic and time-related variability in depression risk. Such approaches can account for spatial clustering and incorporate variables not available in the current dataset [53]. Another limitation of this study is the inability to account for residential mobility [54]. The Riskesdas 2018 dataset does not include information on participants’ residential history or duration of stay in their current location. As a result, some individuals may have recently relocated to districts with different healthcare infrastructure or environmental characteristics. This introduces the potential for relocation bias, particularly if recent movers differ systematically from long-term residents in ways that affect depression risk. Future studies incorporating longitudinal or migration data would be better positioned to examine the influence of place-based factors on mental health outcomes.

Finally, the timing of the surveys used in this study differ slightly. For example, Riskesdas was carried out in 2018, Risnakes in 2017, the Happiness index in 2017, GDRP used in 2018 and population which was referred to assess the density of health care based on population census projections in 2010 because the census was carried out every 10 years. While it can impact research results, it can also enhance them [55]. This is achieved through a careful data harmonization process, which must be carried out carefully [56].

The results of this research strengthen the theory that the factors that influence depression are factors at the individual level, so depression control programs must consist of increasing screening at the individual and family levels. The program can be age-stratified, for example, groups of teenagers, adults, and old people or in the form of groups of school children, office workers, housewives or groups of elderly people and people who live alone and isolated.

The program output targets must also vary between groups. For example, for groups of school children, the aim is to ensure that students are still able to complete their education and achieve well, and for older people to avoid the risk of accidents, falls, suicide and so on [57, 58]. Likewise, workers are expected to live productive lives.

Our findings indicate that individual health conditions, alongside socioeconomic factors, are key predictors of depression. Therefore, policy recommendations should include expanding access to healthcare for those with chronic illnesses or disabilities, as these groups face heightened depression risks. Strengthening community-based mental health services would ensure that individuals with comorbid conditions receive appropriate care. Integrating depression screenings into primary healthcare, particularly for individuals with pre-existing health conditions, could facilitate early detection and intervention. Through Minister of Health Number 19 in 2024 concerning PHCs, the Indonesian government states that PHCs organise promotive, preventive, curative and rehabilitation programs for individuals and communities in their working areas. Specifically, there are several examples of mental health programs at PHCs by implementing community mental health nursing and training mental health cadres [59, 60]. Meanwhile, further research in Indonesia could include variables that have not been included in this research, such as biological factors, stressors and life events, environmental conditions, and current health conditions.

## Conclusion

Determinants at the individual level are an important factor in depression, and these results strengthen previous research, which also states that the individual level is more influential than other contextual levels. The study’s results may offer insights for improving individual-level mental health programs, thereby fostering resilience to depression through enhanced effective prevention programs in mental health. Economic factors at the individual level play a role in the incidence of depression.

## Electronic supplementary material

Below is the link to the electronic supplementary material.

Supplementary Material 1

## Data Availability

Data are available upon official request. The data set (RISKESDAS) can be accessed with approval by Director General of Health Policy Agency, Ministry of Health, Republic of Indonesia, at https://layanandata.kemkes.go.id/request.

## Abbreviations

MINI: Mini International Neuropsychiatric Interview
LMICs: Low- And Middle-Income Countries
DALYs: Disability-Adjusted Life Years
CNS: Central Nervous System
HPA: Hypothalamic-Pituitary-Adrenal
MRI: Magnetic Resonance Imaging
Riskesdas: Riset Kesehatan Dasar
Risnakes: Riset Tenaga Kesehatan
SPTK: Survei Penilaian Tingkat Kebahagiaan
CBS: Central Bureau of Statistics
GDRP: Gross Domestic Regional Product
ICD-10: International Classification of Diseases and Related Health Problems-10
PCA: Principal Component Analysis
GP: General Practitioners
PHC: Primary Health Care Centre
MOR: Median odds ratio
ICC: Intra class correlation
NEF: New Economic Foundation
