# Supplementary material for: Individual and area-level factors associated with depression in Indonesia: a multilevel analysis using the 2018 national basic health research
Source: BMC Public Health. 2025 Oct 2;25:3298. doi: 10.1186/s12889-025-23434-4 (PMC12492620; doi:10.1186/s12889-025-23434-4)
Supplement: Supplementary file 1 — Supplementary Material 1 [file 12889_2025_23434_MOESM1_ESM.docx]

Appendix 1. Depression MINI : the depression module of MINI ICD-10 translated into Indonesian.

| A1 | **In the past two weeks:** |  |  |  |  |
| --- | --- | --- | --- | --- | --- |
| a | Have you been consistently sad, depressed or down, most of the day, nearly every day? |  | No | Yes |  |
| b | Have you been most of the time less interested in most things or less able to enjoy the things you used to enjoy? |  | No | Yes |  |
| c | Did you feel tired or without energy, most of the time? |  | No | Yes |  |
|  | **IF LESS THAN 2 YES in A1** |  |  |  | 🡺  **STOP** |
| A2 | **In the past two weeks, when you felt depressed / uninterested / tired:** |  |  |  |  |
| a | Did your appetite change significantly or did your weight increase or decrease without trying intentionally? |  | No | Yes |  |
| b | Did you have trouble sleeping nearly every night (difficulty falling asleep, night or early awakenings, hypersomnia)? |  | No | Yes |  |
| c | Did you talk or move more slowly than normal, or were you fidgety, restless or having trouble staying still? |  | No | Yes |  |
| d | Did you lose your self-confidence, or did you feel worthless or even inferior to other people? |  | No | Yes |  |
| e | Did you have feelings of self-reproach or guilt? |  | No | Yes |  |
| f | Did you have difficulty thinking or concentrating, or did you have trouble making decisions? |  | No | Yes |  |
| g | Did you consider hurting yourself, feel suicidal, or wish that you were dead? |  | No | Yes |  |
|  | ARE **4** OR MORE ITEMS SINCE A1 CODED **YES**? |  | **F 32. *DEPRESSIVE EPISODE*** | | |
